# Activated leukocyte cell adhesion molecule (ALCAM) facilitates trafficking of HTLV-1 infected lymphocytes through the blood brain barrier

**DOI:** 10.1186/1742-4690-12-S1-P64

**Published:** 2015-08-28

**Authors:** Florent Percher, Céline Curis, Patricia Jeannin, Danielle Seilhean, Pierre-Olivier Couraud, Olivier Gout, Antoine Gessain, Pierre-Emmanuel Ceccaldi, Philippe V Afonso

**Affiliations:** 1 Unité EPVO, Institut Pasteur, CNRS UMR 3569, Paris, France; 2 Laboratoire de Neuropathologie, Hôpital de la Salpêtrière, Paris, France; 3 Institut Cochin, INSERM U1016; CNRS, UMR 8104; Université Paris Descartes, Sorbonne Paris Cité, Paris, France; 4 Cellule Pasteur, Université Paris Diderot, Sorbonne Paris Cité, Paris, France

## 

HAM/TSP is a neurodegenerative disease that develops upon infiltration of HTLV-1-infected lymphocytes into the central nervous system (CNS). Physiologically, the CNS is protected and isolated from the immune system by a structure called blood-brain barrier (BBB). Thus HTLV-1-infected lymphocytes have the capacity to cross the blood-brain barrier (BBB). The mechanisms of such crossing are still poorly understood. In the context of multiple sclerosis and neuro-AIDS, the Activated Leukocyte Cell Adhesion Molecule (ALCAM) was shown to amplify leukocyte extravasation. In the earlier, ALCAM is over expressed on the BBB endothelium, whereas in the latter ALCAM expression is increased on HIV-1-infected monocytes. We therefore studied the possible role of ALCAM in extravasation of HTLV-1 infected lymphocytes. We demonstrated by FACS that ALCAM is over expressed both on HTLV-1-infected lymphocytes cell lines and on primary cells from HTLV-1 asymptomatic carriers or HAM/TSP patients. Via transduction with a Tax-encoding lentiviral vectors, we showed that ALCAM over expression is the consequence of Tax-1-induced NF-κB pathway activation. We finally demonstrated that inhibiting ALCAM with a monoclonal blocking antibody reduces significantly the extravasation of HTLV-1 chronically infected cells through a monolayer of BBB endothelial cells (the hCMEC/D3 model). This study reports a potential role of HTLV-1-induced ALCAM overpression in HAM/TSP pathogenesis.

